# Does Maternal Diet Vary During the Postpartum Period According to Infant Feeding Type? The Observational Study GREEN MOTHER

**DOI:** 10.3390/nu17071136

**Published:** 2025-03-25

**Authors:** Rosa Cabedo-Ferreiro, Azahara Reyes-Lacalle, Judit Cos-Busquets, Margalida Colldeforns-Vidal, Liudmila Liutsko, Rosa García-Sierra, Mª-Mercedes Vicente-Hernández, Miriam Gómez-Masvidal, Laura Montero-Pons, Gemma Cazorla-Ortiz, Pere Torán-Monserrat, Gemma Falguera-Puig, The GREEN MOTHER Group

**Affiliations:** 1 Reproductive and Sexual Healthcare (ASSIR) Granollers, Catalan Institute of Health (ICS), 08400 Granollers, Spain; mcolldeforns.ics@gencat.cat; 2 Research Group on Sexual and Reproductive Healthcare (GRASSIR) (2021-SGR-793), 08007 Barcelona, Spain; areyesl.ics@gencat.cat (A.R.-L.); mvicenteh.mn.ics@gencat.cat (M.-M.V.-H.); miriamgomez.girona.ics@gencat.cat (M.G.-M.); lmontero.ics@gencat.cat (L.M.-P.); gcazorlao.germanstrias@gencat.cat (G.C.-O.); gfalguera.mn.ics@gencat.cat (G.F.-P.); 3 Reproductive and Sexual Healthcare (ASSIR) Sabadell, Catalan Institute of Health (ICS), 08203 Sabadell, Spain; juditcos.mn.ics@gencat.cat; 4 Metropolitan North Research Support Unit (USR), Jordi Gol i Gorina University Institute Foundation for Primary Health Care Research (IDIAP), 08303 Mataró, Spain; rgarciasi.mn.ics@gencat.cat; 5 Multidisciplinary Research Group in Health and Society (GREMSAS) (2021-SGR-0148), 08007 Barcelona, Spain; 6 Reproductive and Sexual Healthcare (ASSIR) Badalona–Sant Adrià, Catalan Institute of Health (ICS), 08930 Sant Adrià del Besós, Spain; 7 Reproductive and Sexual Healthcare (ASSIR) Mataró, Catalan Institute of Health (ICS), 08302 Mataró, Spain; 8 Reproductive and Sexual Healthcare (ASSIR) Santa Coloma de Gramenet, Catalan Institute of Health (ICS), 08921 Santa Coloma de Gramenet, Spain; 9 Faculty of Nursing, Universitat de Barcelona, 08907 L’Hospitalet de Llobregat, Spain; 10 The Institute for Health Science Research Germans Trias i Pujol (IGTP), 08916 Badalona, Spain; 11 Department of Medicine, Faculty of Medicine, Universitat de Girona, 17003 Girona, Spain

**Keywords:** maternal nutrition, postpartum period, breastfeeding, energy and nutrient intake, maternal nutritional physiological phenomena

## Abstract

**Background**: Breastfeeding mothers have an increased demand for nutrients, including increased intake of certain nutrients, and are recommended to consume a theoretical 500 extra kilocalories (kcal), follow a varied diet, and increase protein, carbohydrate, omega-3, iron, and vitamin D intake. **Objectives**: We sought to analyze mothers’ energy and nutrient intake and food habits during the postpartum period 6 weeks after delivery and to identify whether there are any differences between breastfeeding and non-breastfeeding mothers. **Methods**: This was a cross-sectional, multicenter observational study at seven sexual and reproductive healthcare centers in the Metropolitan North area of Barcelona (Spain). The sample comprised 393 women who responded to an infant feeding questionnaire and 24 h diet recall (24 HR). We used frequencies and medians for descriptive analysis as well as the chi-squared and Kruskal–Wallis tests for the bivariate analysis. **Results:** Mean energy intake was lower than the recommendations in 57% of participants. Mothers who exclusively breastfed consumed a median of 201 kcal more than non-breastfeeding mothers, although this was not significant. The intake of fatty acids and micronutrients, such as iron, calcium, magnesium, and especially vitamin D, was insufficient. Breastfeeding mothers consumed significantly more polyunsaturated fatty acids (PUFAs) (*p* = 0.0297): 15.4 g vs. 12.7 g per day. **Conclusions**: There are no significant differences between the diet of breastfeeding and non-breastfeeding women, except for PUFA intake. A general insufficient intake of the analyzed micronutrients was observed. Educational campaigns and dietary guidance from health professionals are a priority.

## 1. Introduction

During the postpartum period (understood as the first six weeks following childbirth), mothers have increased, with specific nutritional needs compared to other women of childbearing age due to recovery after childbirth and to ensure the infant’s normal development while breastfeeding [1]. Leading health organizations, such as the World Health Organization (WHO), the United Nations Children’s Fund (UNICEF), and the American Academy of Pediatrics (APP), recommend exclusive breastfeeding during the first six months of a newborn’s life, followed by continued breastfeeding with complementary foods for at least two years [2,3,4,5]. Breastfeeding mothers require increased nutrients. Breast milk contains all the nutrients necessary for the baby up to six months of age, except for vitamin D and vitamin K [6].

Energy stores are mobilized, nutrients are depleted during pregnancy, 500 to 1000 mL of blood is lost during childbirth, and weight is lost during the postpartum period. While non-breastfeeding mothers may have the same nutritional needs as other women of childbearing age, they require a healthy and balanced diet for their recovery during the immediate postpartum period [7].

The WHO [8], the Food and Agriculture Organization of the United Nations [7], and the European Food Safety Authority [9] estimate that the recommended average energy intake for women of childbearing age is 1750–2570 kcal/day depending on physical activity. The theoretical extra daily energy required by a breastfeeding woman was estimated to be 2.1 mega joules (MJ/day) (500 kcal/day) [7,9], based on a required 2.8 MJ/day (670 kcal/day) for breast milk production and the mobilization of 0.72 MJ/day (170 kcal/day) of energy stores from maternal tissue [9]. However, the National Academy of Sciences estimates that breastfeeding women require an additional 330 kcal/day based on an increased energy intake for milk production of 500 kcal minus physiological weight loss of 170 kcal [10].

The recommended macronutrient distribution range for a woman of childbearing age is 45–60% carbohydrates (CHO), 12–15% proteins (P), and 20–35% fat (FA) [9]. Regarding the nutritional requirements of breastfeeding women, CHO intake should be increased by an estimated 210 g/day, preferably in the form of complex CHOs, including whole grains, legumes, and tubers [11]. P intake should be increased by 15–19 g/day [12]. There is no recommendation to increase total FA consumption; breastfeeding women are recommended to consume as little saturated FA (sat FA) as possible, or less than 10% of total daily intake, equivalent to that of a woman of childbearing age. As for poly- and mono-saturated fats, the recommendations include increasing omega-3 up to 350–450 milligrams (mg) daily [9] as well as limiting the consumption of PUFAs to 6–11% of total energy consumed [13] and monounsaturated fatty acids (MUFA) to 4.5% of daily food consumption, which includes virgin olive oil, oily fish, nuts, seeds, etc. [9]. The WHO recommends avoiding the consumption of trans fats as they are associated with increased risk of coronary heart disease (CHD) events and mortality [14]. Breastfeeding women are recommended to consume foods containing omega-3, especially docosahexaenoic acid (DHA), to reach a minimum of 200 mg/day [15], the equivalent of one to two portions of fish per week [16,17]. DHA levels have been shown to be positively associated with the intellectual and central nervous system and the retinal development of the infant [18] in neurological examinations [19], as well as the prevention of chronic disease in adults [20]. There is a positive correlation between PUFA levels, especially DHA, in the mother’s diet and breast milk [13,21,22,23]. In contrast, no significant association between maternal DHA/EPA supplementation and infant cognitive performance has been identified [24,25,26]. However, omega-3 supplementation has been linked to a lower incidence of associated allergies in babies [27]. A recent systematic review shows that maternal body mass index and macronutrient intake influence the fatty acid composition of breast milk, affecting infant growth and metabolic and cognitive development [27].

The daily recommendations for micronutrients during the postpartum period are 15 µg/day of vitamin D, 950 mg of calcium, 300 mg of magnesium, and 16 mg of iron, although this should be higher if the mother had anemia during pregnancy or experienced a hemorrhage during childbirth [9]. The levels of calcium [28], phosphorus, and magnesium in breast milk are independent of intake. By contrast, vitamins A, D, K, C, B1, B6, and B12 do depend on and respond to supplementation [1]. Some breastfeeding mothers choose to take supplements to compensate for potential micronutrient deficiencies, as some are difficult to obtain through food alone [6,29,30].

Both nutritional deficiencies and excesses in postpartum women can impact the quantity of milk and, to a lesser extent, its quality [6,30,31] and affect maternal and fetal health in the medium to long term [32]. The prevalence of nutritional deficiencies varies according to culture, eating habits, and socioeconomic status [6]. Maternal nutrition is sometimes believed to differ considerably from the Mediterranean diet. A gradual decline in this type of diet has been observed during pregnancy and the postpartum period [33]. Since our population is heterogeneous, we must consider social, economic, and cultural factors when intervening in breastfeeding and feeding [6,34].

In light of these theoretical data on maternal nutrition, we aim to describe the energy intake, nutrition distribution, and food habits of mothers in the Metropolitan North area of Barcelona 6 weeks post partum and identify whether there are differences between those who breastfeed and those who do not.

This article is part of a larger project that aims to research breastfeeding, healthy eating, and sustainability: the Green Mother project [35].

## 2. Materials and Methods

### 2.1. Design and Study Setting

This was a multicenter, cross-sectional study with consecutive sampling of pregnant women in their third trimester and postpartum mothers at their first postpartum checkup receiving prenatal care at a Reproductive and Sexual Health Care unit (ASSIR, from the Spanish Atenció a la Salut Sexual y Reproductiva). The seven ASSIR units of the Barcelona Metropolitan North area participated. These are located in the primary care facilities of the public health system in Catalonia (Spain).

Data were reported by the women recruited and collected by midwives from November 2022 to April 2023.

### 2.2. Sampling and Study Population

A random sample of 402 participants is sufficient to estimate an expected population percentage of approximately 50%, with a 95% confidence interval and a precision of +/−5%. The percentage of necessary replacements has been predicted to be 10% (software: GRANMO, version 7.11, n.d.).

### 2.3. Inclusion and Exclusion Criteria

The inclusion criteria were voluntary agreement (via signed informed consent) to participate in this study; being pregnant in the third trimester or a postpartum mother at their checkup 7 to 14 days post partum; and being at least 16 years old. The exclusion criteria were language barriers (not speaking Catalan or Spanish) that impeded informed consent and data collection as well as non-continuity (not being sure if they could attend the postpartum follow-ups in the corresponding primary attention center participating in this study).

### 2.4. Measurements

The midwives filled out the study variables on the Research Electronic Data Capture (REDcap) platform, a secure online application [36]. Three questionnaires were designed to collect sociodemographic, clinical, and maternal diet variables. Some of these data were already available in the Catalan electronic clinical record program (eCAP, Estació Clínica d’Atenció Primaria). The ICS (Catalan Health Institute) is responsible for keeping the data secure.

Questionnaires:

a. Questionnaire on the sociodemographic data of mothers: age, education level, religion, place of birth, paid work, and social support (maternity and paternity leave and duration thereof).
b. Questionnaire on clinical data: maternal pathologies and type of infant feeding at one month of life: exclusive breastfeeding (EBF), mixed breastfeeding (MBF), or formula feeding (FF). Type of diet followed by the mother: vegetarian, vegan, gluten-free, for allergies, therapeutic, or no special diet. Use of dietary supplements.
c. Twenty-four--hour dietary recall (24 h) method described by Ferrari [37]: To monitor the mother’s diet, a “24-h food register” was used in this study [37]. The participants filled out the template with a 24 h count before the visit with the midwife, and data about the characteristics of the three main meals and snacks, food, drinks, condiments, portions, type of cooking, and food preservation were collected. A visual food atlas with portions of the different food groups was used to help complete the survey. The Food Atlas guidelines for portions were adapted from the Roche Guide and Photographic Atlas of the Spanish Agency for Food Safety and Nutrition [38,39]. Finally, the dietary 24 h was revised together with midwives during the postpartum visit at the primary care center. Midwives were trained previously on the application of this method.

### 2.5. Procedures

The principal investigator trained the recruiting midwives in how to complete the questionnaires on the REDcap [36] platform and fill out the 24 h register.

1. Postpartum checkup after 7 to 14 days:
  a. The participant was given a printout to record the 24 h. Using the aforementioned atlas, they were instructed on how to record their diet over the course of an entire day.
  b. The sociodemographic and clinical data from the questionnaire were recorded
  c. The feeding type was recorded upon discharge from the hospital.
2. Postpartum checkup after 6 weeks:
  a. The participant turned in the completed 24 h, and it was finally completed and revised jointly with the midwife. Afterwards, it was registered on the REDcap platform.
  b. The feeding type one month post partum was recorded.

### 2.6. Data Analysis

To assess adequate intake levels, we based our calculations on EFSA’s dietary reference values (DRVs) for macro- and micronutrients for breastfeeding women in Europe [9].

Energy intake was estimated in kcal. The percent of energy derived from macronutrients was calculated for P, CHO, and FAs. Total FAs were subdivided into sat FAs, MUFAs, and PUFAs and were measured in grams. Regarding micronutrients, iron (Fe), Calcium (Ca), and magnesium (Mg) were analyzed in mg, and Vitamin D (Vit D) was analyzed in micrograms.

The program Agribalyse v3.1 [40] was used to obtain these data for foods and Ciqual1 [41] for nutrients.

A univariate analysis was used to perform descriptive statistics of sociodemographic, clinical, and nutritional data. A bivariate analysis (chi-squared test for categorical variables and the Kruskal–Wallis test for continuous variables) was performed to detect (1) the most relevant sociodemographic, clinical, and diet-type factors related to dietary energy intake and (2) differences in the consumption of macro- and micronutrients and total energy depending on infant feeding type (EBF, MF, and FF) in terms of medians for each group and frequencies represented by the consumption groups.

### 2.7. Ethics

All participants gave written informed consent after having received complete information about the study.

This project was approved by the Research Ethics Committee of the Jordi Gol i Gorina University Institute Foundation for Primary Health Care Research (IDIAP) under code 22/101-P dated 22 February 2023. This protocol follows the tenets of the Declaration of Helsinki.

## 3. Results

### 3.1. Sociodemographic, Clinical, and Infant Feeding Type Data

A total of 429 women were recruited, of which 393 filled out the questionnaire on feeding type and the 24 HR for maternal diet. The mean age of the postpartum women was 33 years (SD 5.18), 29% were born outside of Spain, and 44% had completed university studies. At one month postpartum, 399 participants responded about infant feeding type: 254 (63.6%) reported that they practiced EBF, 91 (22.8%) practiced MF, and 54 (13.5%) practiced FF.

Table 1 shows the sociodemographic and clinical results. In the bivariate analysis between sociodemographic and clinical factors with energy consumed 6 weeks post partum, no statistically significant differences were observed (*p* > 0.05). The only factor that was marginally statistically significant (chi2 = 5.68; *p* = 0.058) was having a partner with maternity/paternity leave to care for the baby. Of the mothers whose intake was below 1750 kcal, 85.2% had partners who were on parental leave from work.

The bivariate analysis between infant feeding type and vitamin intake showed that breastfeeding mothers consume more vitamin supplements (EBF 72%; MF 65%) than non-breastfeeding mothers (FF 37%), representing a statistically significant difference (*p* < 0.001).

### 3.2. General Description of Maternal Diet Type and Consumption Habits

The majority of participants (94.79%, or 364) did not follow a special diet. Of the rest, six (1.56%) followed a gluten-free diet; five (1.3%) followed a vegetarian diet; five (1.30%) followed a therapeutic diet (diabetes, hypertension or dyslipidemia, etc.); and four (1.04%) followed a special diet due to food allergies.

Produced or ready-to-eat (manufactured product that does not required cooking to be ingested) and uncooked foods were used the most frequently for the three main meals (80–83%), followed by pre-prepared (9–12%), frozen (5–7%), and conserved foods (1.8% canned and 1.5% in glass containers). Regarding cooking methods, 53% of food products were consumed raw or handled minimally by participants; 16% were boiled; 12% were baked; 9% were grilled; 9% were fried; and 2% were steamed. Regarding food products, the most frequently consumed were bread (29%), dairy products (12%), and fruit (10%) for breakfast; cooked vegetables (12%), chicken (10%), fruit (9%), rice (7%), and potatoes (7%) for lunch; and dairy products (13%), cooked vegetables (9%), bread (9%), eggs and their derivatives (8%), and vegetable soups (7%) for dinner. As for dressings, oil and vinegar were the most frequently used, at 60% for lunch and 53% for dinner, while sauces accounted for 20% and 9%, respectively. Lastly, butter represented 1% of the total dressing.

Regarding beverages, the most frequently consumed were animal milk (54.45%) for breakfast and water for lunch (89%) and dinner (92%). Alcohol was consumed by three participants, one from each type of infant feeding. Sweetened beverages (soft drinks and bottled juice) represented 3.4% of drinks at breakfast, 10.54% at lunch, and 6.22% at dinner, with no significant differences across the different infant feeding types.

### 3.3. Quantitative Results of Maternal Diet in the Postpartum Period (4–6 Weeks): Energy, Macro- and Micronutrients

Energy intake by participants who practiced EBF was 1705 kcal, while it was 1504 kcal for those who practiced FF. This 201 kcal difference was not statistically significant (Table 2).

In our study, the ratio of CHOs was 56%, 61%, and 60% for FF, MF, and EBF, respectively; the ratio of P was 23% for all feeding types; and the ratio of FAs was 21%, 16%, and 17% for FF, MF, and EBF, respectively. In PUFA consumption, the difference was statistically significant between the infant feeding type groups (*p* = 0.0297). Breastfeeding mothers (EBF and MF) consumed more PUFAs than non-breastfeeding mothers (chi2 = 6.95, *p* = 0.0084 for EBF and chi2 = 4.11, *p* = 0.042 for MF, respectively). This difference was not statistically significant for the consumption of PUFAs by mothers practicing EBF and MF. Table 2 presents the estimated values for macronutrients (P, CHOs, and saturated/mono and poly FAs) and energy consumed during the 24 HR of mothers (not including drinks).

As for micronutrients, we analyzed Fe, Vit D, Ca, and Mg. The median intake of Fe was 7.9 mg per day; 79% of participants consumed less than the recommended dietary intake (RDI). The median intake of Vit D was 2.3 μg; 13% of users met the RDI. As for Ca, the median intake was 446 mg, with only 8% of participants reaching the RDI. Lastly, the median Mg intake was 169 mg, with 74% of participants consuming less than the RDI. Table 3 presents the results of the micronutrients by maternal diet type and classified according to the RDI.

Table 4 and Table 5 show the distribution of macro- and micronutrients according to the type of maternal diet. The least energy consumption (805 kcal) was observed in five women with a therapeutic diet (diabetes, hypertension, dyslipidemia…), followed by women with a vegetarian diet (1475), whereas the rest had energy consumption expressed in median values (1673–1942) (Table 4).

The women with vegetarian diets obtained 14.2 mg in Fe consumption as the median (n = 5), though the confidence interval (95%CI) had huge variation [2.9–21.9] (Table 5). Women with a therapeutic diet (diabetes, hypertension, dyslipidemia...) had 5.7 [3.9–22.7] mg of Fe consumption and 272 [121–419] mg of Ca consumption (Table 5).

## 4. Discussion

Few studies focus on maternal nutrition during the postpartum period. Most assess the impact of maternal diet on breastfeeding.

Our study sample comprised 393 women. Considering the collection of a day’s diet, energy intake was insufficient in 57% of participants. Breastfeeding mothers consumed a median of 201 kcal more than non-breastfeeding mothers. Participants’ intake of micronutrients and healthy FA was insufficient. However, breastfeeding mothers consumed significantly more PUFAs.

In the bivariate analysis between sociodemographics and energy consumed 6 weeks post partum, the only factor that was marginally statistically significant was whether or not the mother had a partner on parental leave to care for the baby. These results merit in-depth exploration using a qualitative method to interpret them. Other studies identify women at risk of malnutrition who require nutrition intervention, such as smokers and those with less education [42].

Our results show a high consumption of produce and minimally processed food (81.7%), which is in line with the recommendations. Although participants consumed fried foods at a moderate rate (9% of all meals), the recommendation is to avoid them as they are high in saturated fats and trans fats that increase the risk of pathologies, such as cardiovascular diseases and colorectal cancer [43,44]. In the CastelLact Study, frying was the least common cooking method, at just 15.4% [45].

The mean consumption of carbonated and sweetened beverages was 7% at main meals, which can be considered elevated, and did not vary between breastfeeding and non-breastfeeding mothers. Regular consumption (1–2 doses/day) of sweetened beverages is associated with an increased risk of type 2 diabetes [46]. Regarding alcohol, it was consumed by one woman practicing EBF and another woman practicing MF. We suspect there is some degree of underreporting due to the social and health perceptions associated with consuming toxic substances during pregnancy and breastfeeding. In a study conducted by Blasco-Alonso in Malaga, a mean of 17–22% of pregnant women consumed alcohol. Public health campaigns and medical guidance are essential to redirect this situation, much like is done for the issue of smoking [47]. Among the reasons for consuming alcohol while breastfeeding was the belief that alcohol stimulates breast milk production, unclear guidance from physicians, unawareness of the risks of infant exposure, and to boost mood and celebrate events [48]. Alcohol consumption during the breastfeeding period significantly compromises a child’s development [49].

From the quantitative assessment of diet, we observed that the median energy intake was 1504 kcal in women practicing FF, 1554 kcal in women practicing MF, and 1705 kcal in women practicing EBF. The daily energy count was lower than recommended for a healthy diet in all groups [9], bearing in mind that, in general, postpartum women are only minimally physically active and thus do not need more than 1750 kcal daily. Despite this, 69% of women practicing FF still did not reach the minimum required energy intake.

Breastfeeding mothers are recommended to increase their daily intake by 230–500 extra kcal, but 53% do not even reach the minimum recommendation. In our study, the median difference in kcal between breastfeeding and non-breastfeeding women was 201 kcal. Some studies report that eating disorders rise during the postpartum period due to aesthetic pressure and the desire to lose weight quickly by consuming fewer calories than necessary [50,51,52]. There are other concerns as well, such as inadequate energy intake due to health conditions and poor food choices, not having time to eat, and excessive consumption of sweets and caffeine. In a study of 30 women in Madrid (Spain) that examined the nutritional status of mothers in the first month of breastfeeding, participants consumed 1870 kcal/day [53]. In 2021, Claude Billeaud obtained similar results, indicating a low-calorie diet (1992 kcal/day) [54].

Restrictive diets of less than 1800 kcal per day can affect maternal energy stores. Consuming less than 1500 kcal/day can result in fatigue and diminish the volume and composition of breast milk and, therefore, the nutrients the infant receives, thus impacting their health [6]. However, when the body lacks energy, it reacts by increasing the secretion of prolactin to maintain the production of breast milk, although the effect on long-term production is unknown [12].

Weight loss during breastfeeding generally does not affect the quantity or quality of breast milk, but maternal deficiencies in magnesium, vitamin B6, folate, calcium, and zinc have been reported during breastfeeding [1].

In our sample, the distribution of macronutrients was 23% P, 60% CHOs, and 17% fat. From these data, we observe excessive protein intake, or 8% more than recommended, at the expense of sufficient fat intake for a balanced diet. This is likely the cause of the low energy count.

The CastelLact Project in Spain analyzed 26 breastfeeding women with a mean energy intake of 2575.88 kcal/day (±730.59 SD), comprising 45% CHOs and 40% FAs, including 37.16 g ± 10.43 of sat FAs. Breastfeeding mothers partially met nutritional targets, but they consumed excess sat FAs and insufficient calcium, iodine, vitamin D, E, and folic acid [45]. The total FA and total energy intake were much higher than in our study (with a sample of 393 participants), and yet micronutrient deficiencies were still observed.

Median P consumption was 78.7 g per day. There is no established maximum protein amount, although if energy consumption is insufficient, as in our sample, P can be used for energy production [6].

CHO consumption was slightly higher, including a number of foods rich in simple and refined CHOs, which exceeds the recommended maximum of 10%. The rate of consumption of fruit, at 9%, was adequate according to recommendations. Conversely, there is evidence of a low intake of vegetables, greens, and legumes. Other authors also report insufficient intake of these foods during pregnancy and breastfeeding, as well as of nuts, eggs, and fish, alongside excess consumption of ultra-processed meats, pastries, and sweets [15].

FA and micronutrient intake have been linked to their content in breast milk [55]. A systematic review published in 2024 on maternal diet and breastfeeding shows a link between the mother’s diet and fatty acids in breast milk [23]. In our study, a similar consumption of animal versus vegetable fats is observed. However, the intake of foods rich in MUFAs and PUFAs, such as olive oil, nuts, and oily fish, is generally low, as was pointed out in the narrative review of Carretero-Krug [13]. In addition, 20% of participants consume sauces as a condiment rather than olive oil. This may impact the omega-3 and -6 and fat-soluble vitamin content in breast milk [6,31]. When comparing consumption across infant feeding types, breastfeeding mothers consumed significantly more PUFAs. Gila-Diaz et al. obtained similar results [53] Some authors recommend DHA supplementation from pregnancy onwards to prevent potential long-term consequences for the baby [54]. There should be greater emphasis on promoting healthy FA intake, with an increase in MUFAs and PUFAs and a decrease in sat FAs and trans FAs, especially in women who wish to breastfeed.

Similarly, we found that iron intake was deficient in more than 79% of women and across all groups, regardless of infant feeding type. Vegetarian and vegan mothers consume foods with non-heme iron, so proper food combination should be encouraged to promote absorption [56]. Iron supplementation is recommended for women who are anemic at the time of delivery or who experience postpartum hemorrhage.

The median vitamin D intake was 2.3 µg (2.2–2.5 µg), far from the 15 µg daily requirement of 15 µg. This deficiency is considered widespread in the European population, and systematic supplementation is recommended to avoid musculoskeletal and other health problems in women. Bluefish should be recommended, as it contains vitamin D and omega-3 [57]. This is in addition to the recommended 15–20 min/day of sun exposure [6]. Likewise, vitamin D transfer to breast milk is limited, which is why the recommendation in Spain is that all breastfed newborns also receive oral supplementation [6,54]

As for calcium, intake was deficient in 92% of postpartum women, with a median below even half of the recommended amount. Low consumption of dairy products, bony fish, broccoli, raw nuts, and sesame seeds has been identified, which could help increase calcium intake [58]. Calcium deficiency primarily affects the mother’s long-term bone health [59]. Supplementation is not considered necessary, but dietary guidance is needed to correct such a deficiency [60].

Magnesium was yet another mineral our sample did not consume enough of, with a mean intake of 169 mg, while the estimated required amount is 300 mg [9]. A diet rich in leafy green vegetables, such as a vegetarian diet, should be recommended to meet daily magnesium requirements.

As for vegetarian, vegan, and therapeutic diets, they often present a significant deficit of FAs and micronutrients. According to Karcz, specific monitoring and nutrition advice is necessary for any diet that requires restricting some food type [56]. Although most breastfeeding women do not consume enough micronutrients, 34% do not take any vitamin supplements. Lastly, diets based on the Harvard Healthy Eating Plate [61] guidelines should be recommended, which include plenty of fruits and vegetables; reduced consumption of meat (especially red meat); increased intake of fish, legumes, and nuts; and limited consumption of ultra-processed and sugary products.

### Limitations and Future Studies

Migrants with language barriers or illiteracy could not be represented in our study due to the impossibility of communicating with them in writing or verbally. There is a portion of the population that receives private health care and does not use the public system, so this study is not fully representative of the whole population.

Ideally, the 24 h recall nutrition survey would be collected over three separate days to allow for individualized analysis. One-day recall it is not a long enough time to accurately estimate some vitamin and mineral intake. However, this is difficult to replicate without a high rate of participants lost to follow-up due to time constrictions in the postpartum period (dedicated to mother’s recovery and infant care). There have been studies with a consensus to collect data from a single day and, thanks to our sample size of 393 women, the average diet is represented. There are not many studies with this sample size and with data on diet and food intake.

Dietary supplements (vitamins and micronutrients) consumed by the participants of this study were not counted in detail due to the huge dispersion of existing products. In future studies, it is recommended to consider these limitations and explore more details collected together with the diet.

## 5. Conclusions

No significant differences were found between the diet of breastfeeding and non-breastfeeding mothers, except for PUFAs, which breastfeeding mothers consumed in marginally greater quantities. There is a general deficit in both total energy intake and healthy FA and micronutrient intake across the entire study population. There is also an excessive intake of refined carbohydrates and simple sugars, mainly from sugary drinks. A personalized approach to healthy nutrition is needed both at pregnancy and postpartum checkups, taking into account the sociocultural, economic, and health profile of mothers.

Professionals must complete ongoing training and stay updated to improve the nutrition of women in the postpartum period and, more significantly, during breastfeeding. Policies to support and protect breastfeeding and community education interventions in healthy nutrition in primary health care are essential.

## Figures and Tables

**Table 1:** Sociodemographic and clinical characteristics of participants (n = 393).

Variables	N	Categories	n	(%)
Age (group)	389	≤25	32	(8.23)
		26–35	220	(56.56)
		≥36	137	(35.22)
Education	327	None or primary	68	(20.80)
		Compulsory secondary	27	(8.26)
		Professional education	87	(26.61)
		University	145	(44.34)
Religion	391	Christian	167	(42.71)
		Muslim	20	(5.12)
		Buddhist	2	(0.51)
		Agnostic	136	(34.78)
		Others	66	(16.88)
Place of birth	392	Spain	280	(71.43)
		North Africa	9	(2.30)
		Sub-Saharan Africa	3	(0.77)
		Europe	16	(4.08)
		Asia	7	(1.79)
		Hindustani region	2	(0.51)
		Central and South America	74	(18.88)
		North America	1	(0.26)
Paid work mother	390	No	59	(15.13)
		Yes	331	(84.87)
Maternity leave	391	No	61	(15.60)
		Yes	330	(84.40)
Paternity/maternity leave partner	388	No	71	(18.30)
		Yes	317	(81.70)
Maternal pathology	393	Diabetes	13	(3.31)
		Hypertension	13	(3.31)
		Gastrointestinal disease	7	(1.78)
		Autoimmune disease	7	(1.78)
		Endocrine disease	27	(6.87)
		Mental disorder	10	(2.54)
		Breast surgery	3	(0.76)
		Others	48	(12.21)
Dietary supplements	391	No	134	(34.27)
		Yes	257	(65.73)

**Table 2:** Macronutrients and energy in maternal diet according to different infant feeding types one month post partum (n = 393).

Variable (Unit)			Total	Infant Feeding Type (FT)			DIF FT (Sign)
				EBF	MF	FF	
Energy (kcal)	Mdn [95% CI]	1672 [1564–1717]	1705 [1617–1812]	1554 [1404–1745]	1504 [1345–1708]	*p* = 0.361	
		***reference level***	***% participants/category***				
		<1750	57	53	61	69	*p* = 0.168
		**1750–2570**	**37**	**42**	**32**	**25**	
		>2570	6	5	7	6	
**CHO (g)**		**Mdn** [95% CI]	278.5 [264.6–306.9]	286.8 [267.2–319.9]	268.1 [247.8–330.7]	265.0 [228.3–347.4]	*p* = 0.880
		***reference level***	***% participants/category***				
		<45%	0	0	0	0	*p* =0.965
		**45–60%**	**25**	**25**	**24**	**25**	
		>60%	75	75	76	75	
**P (g)**		**Mdn** [95% CI]	78.7 [75.7–84.0]	79.08 [75.51–86.54]	78.38 [69.20–87.92]	80.51 [71.25–87.35]	*p* = 0.907
		***reference level***	***% participants/category***				
		<12%	0	0	1	0	*p* = 0.254
		**12–20%**	**27**	**28**	**30**	**20**	
		>20%	73	72	69	80	
**FA total (g)**		**Mdn** [95% CI]	56.4 [52.6–58.9]	57.3 [53.2–61.5]	54.4 [48.6–60.0]	51.0 [45.2–62.1]	*p* = 0.232
		***reference level***	***% participants/category***				
		<20%	67	66	68	71	*p* = 0.483
		**20–35%**	**29**	**28**	**28**	**29**	
		≥35%	4	6	3	0	
**FAsat (g)**		**Mdn** [95% CI]	19.8 [18.8–21.4]	20.4 [18.8–22.03]	19.0 [16.5–21.4]	20.6 [18.0–26.4]	*p* = 0.464
		***reference level***	***% participants/category***				
		**<10%**	**92**	**91**	**95**	**92**	*p* = 0.499
		≥10%	8	9	5	8	
**MUFA (g)**		**Mdn** [95% CI]	17.7 [16.1–19.1]	18.0 [16.0–21.1]	17.2 [15.6–19.3]	14.9 13.4–20.2]	*p* = 0.338
		***reference level***	***% participants/category***				
		<4.5%	44	43	47	45	*p* = 0.786
		**≥4.5%**	**56**	**57**	**53**	**55**	
PUFA (g) *		**Mdn** [95% CI]	15.3 [14.4–16.4]	15.4 [14.5–17.2]	15.7 [13.7–17.4]	12.7 [11.0–16.1]	*p* = 0.0297 *
		***reference level***	***% participants/category***				
		**<6%**	71	70	67	**80**	***p* = 0.299**
		**6–11%**	23	23	26	**20**	
		**>11%**	6	7	7	**0**	

Legend: FF: formula feeding; MF: mixed feeding; EBF: exclusive breastfeeding; P: protein; CHO: carbohydrate; FA: fatty acid; FAsat: saturated fatty acid; MUFA: monounsaturated FA; PUFA: polyunsaturated FA. Reference level for macronutrients and total energy is highlighted in grey [9]. * For PUFA recommendation levels, we used the FAO [13].

**Table 3:** Micronutrients in maternal diet according to different infant feeding types one month post partum (n = 393).

Variable (Unit)		Total	Infant Feeding Type (FT)		
			EBF	MF	FF
**Fe (mg)**	**Mdn [95% CI]**	7.9 [7.5–8.7]	8.0 [7.5–8.7]	8.4 [7.2–9.8]	7.1 [6.7–9.4]
	***reference levels:***	***% participants***			
	<16 mg	79	78	78	80
	**≥16 mg**	**21**	**22**	**22**	**20**
**Vit D (µg)**	**Mdn [95% CI]**	2.3 [2.2–2.5]	2.4 [2.2–2.7]	2.2 [2.0–2.6]	2.1 [1.7–2.5]
	***reference levels:***	***% participants***			
	<15 µg	87	87	90	82
	**≥15 µg**	**13**	**13**	**10**	**18**
**Ca (mg)**	**Mdn [95% CI]**	446 [405–510]	470 [424–545]	364 [272–491]	436 [245–604]
	***reference levels:***	***% participants***			
	<950 mg	92	92	96	88
	**≥950 mg**	**8**	**8**	**4**	**12**
**Mg (mg)**	**Mdn [95% CI]**	169 [160–180]	174 [162–189]	172 [150–206]	158 [134–169]
	***reference levels:***	***% participants***			
	<300 mg	74	73	73	78
	**≥300 mg**	**26**	**27**	**27**	**22**

Legends: FF: formula feeding; MF: mixed feeding; EBF: exclusive breastfeeding; Fe: iron; Vit D: vitamin D; Ca: calcium; Mg: magnesium. The micronutrient intake reference level is highlighted in grey [9].

**Table 4:** Descriptive of total energy intake and macronutrients by maternal diet type.

Diet Type	n	Energy (kcal)	Macronutrients (Mdn [95%CI])					
		(Mdn [95%CI])	P (g)	CHO (g)	FA (g)	FA sat (g)	PUFA (g)	MUFA (g)
No Special Diet	364	1673 [1567–1714]	79.5 [75.6–84.7]	279 [265–307]	57 [54–60]	20 [19–22]	15 [14–16]	18 [16–19]
Vegetarian	5	1475 [629–2092]	69.4 [16.8–106.9]	214 [109–374]	40 [14–54]	17 [7–26]	10 [5–18]	11 [3–18]
Celiac	6	1778 [873–2446]	76.9 [52.1–117.6]	329 [119–1000]	60 [19–88]	20 [5–27]	19 [6–25]	26 [6–36]
Food Allergies	4	1783 [1501–2777]	90.1 [60.3–122.5]	312 [264–1000]	56 [39–90]	21 [18–29]	15 [10–24]	20 [12–37]
Therapeutic Diet (Diabetes, Hypertension, Dyslipidemia…)	5	805 [643–1925]	63.6 [37.3–98.3]	183 [63–219]	28 [19–86]	8 [5–34]	12 [6–15]	8 [6–39]
Unespecified	9	1942 [1221–2842]	93.5 [63.6–119.6]	321 [218–565]	44 [25–73]	18 [9–26]	13 [7–32]	13 [6–22]

Legend: P: protein; CHO: carbohydrate; FA: fatty acid; FAsat: saturated fatty acid; MUFA: monounsaturated FA; PUFA: polyunsaturated FA.

**Table 5:** Descriptive of micronutrients by maternal diet type.

Diet Type	n	Micronutrients (Mdn [95%CI])			
		Fe (mg)	Ca (mg)	Mg (mg)	Vit D (µg)
No Special Diet	364	7.8 [7.4–8.6]	450 [407–520]	169 [160–180]	2.3 [2.2–2.5]
Vegetarian	5	14.2 [2.9–21.9]	488 [108–902]	210 [69–437]	2.0 [0.2–17.4]
Celiac	6	7.9 [5.1–18.4]	382 [208–755]	176 [102–424]	3.0 [0.5–16.8]
Food Allergies	4	9.6 [8.7–11.4]	350 [137–526]	149 [72–267]	3.1 [1.4–9.1]
Therapeutic Diet (Diabetes, Hypertension, Dyslipidemia…)	5	5.7 [3.9–22.7]	272 [121–419]	146 [92–488]	2.4 [0.6–15.2]
Not Specified	9	9.6 [7.3–18.0]	548 [227–650]	198 [136–383]	2.0 [1.0–3.3]

Legend: Fe: iron; Vit D: vitamin D; Ca: calcium; Mg: magnesium.

## Data Availability

The original contributions presented in the study are included in the article, further inquiries can be directed to the corresponding authors.
